# Fully Integrated MEMS Micropump and Miniaturized Mass Flow Sensor as Basic Components for a Microdosing System †

**DOI:** 10.3390/mi15121404

**Published:** 2024-11-21

**Authors:** Martin Seidl, Gabriele Schrag

**Affiliations:** Department of Electrical Engineering, TUM School of Computation, Information and Technology, Technical University of Munich, 80333 Munich, Germany; schrag@tum.de

**Keywords:** MEMS, integrated, microfluidics, micropump, electrostatic actuation, electrostatic pull-in, mass flow sensor, microdosing

## Abstract

Despite major advances in the field of actuator technology for microsystems, miniaturized microfluidic actuation systems for mobile devices are still not common in the market. We present a micropump concept and an associated mass flow sensor design, which, in combination, have the potential to form the basis for an integrated microfluidic development platform for microfluidic systems in general and microdosing systems in particular. The micropump combines the use of active valves with an electrostatic drive principle for the pump membrane and the valves, respectively. With a size of only 1.86 mm × 1.86 mm × 0.3 mm, the first prototypes are capable of pumping gaseous media at flow rates of up to 110 μL/min. A specific feature of the presented micropump is that the pumping direction is perpendicular to the chip surface. The corresponding flow sensor combines the principle of hot-wire anemometry with a very small footprint of only 1.4 mm × 1.4 mm × 0.4 mm. The main innovation is that the hot wires are fixed inside a through-hole in the substrate of the microchip, so that the flow direction of the fluid to be measured is perpendicular to the chip surface, which enables direct integration with the presented micropump. Detection thresholds of around 10 μL/min and measuring ranges of up to 20 mL/min can be achieved with the first prototypes, without dedicated evaluation electronics.

## 1. Introduction

The rapid miniaturization observed for decades in the field of microsystems, which is being driven, in particular, by mobile devices and mobile communications technology, has been made possible, above all, by the use of silicon technology. However, the ever-increasing packing density and growing functionality of sensor systems mean that the requirements for miniaturized sensors and actuators are becoming increasingly stringent. Sensor technology for mobile devices in many areas, such as position and acceleration measurement, the recording of acoustic signals, or imaging, has found widespread use. Even miniaturized microfluidic environmental sensors [1,2,3] have become available on the market recently. However, despite the advances in the field of actuator technology for microsystems, miniaturized active microfluidic systems are rarely found that meet the requirements for integration into mobile devices or other compact systems [4].

A key element in the development of a new, active microfluidic system, which not only passively utilizes prevalent fluid flows but also is able to generate them itself, is the micropump for driving fluids and gaseous media. Reviews [5,6,7,8,9] of the state of the art in micropumps show that recent developments focus mainly on reliability, miniaturization, and control [10,11]. In [12,13], comprehensive overviews on micropumps that are specifically for use in microdosing systems for drug delivery and in the biomedical field is given and various types of actuation are discussed with a view to specific abilities [14] for different application scenarios. From these overviews, it can be concluded that the most promising and most studied group of micropumps in terms of reliability, downsizing potential, and achievable flow rates is the diaphragm pump. Many different actuation types for the pump membrane were extensively tested, including, but not limited to, piezoelectric actuation [10,15,16,17,18,19,20,21,22,23,24,25,26,27,28,29], electrostatic actuation [30,31,32,33], hydraulic actuation [34,35,36,37], magnetic actuation [38,39], or even the use of electroactive polymers [40,41] or shape memory alloys [42]. However, conventional concepts seem to hit a downsizing limit at edge lengths above 3 mm [10].

Piezoelectric actuation has, so far, shown the most promising results in miniaturized, silicon-based diaphragm pumps, achieving relative flow rates of up to 49 µL/min in relation to a mm² of the chip surface. However, very high electrical operating voltages in the range of 100 V or more, as well as their size, make them unsuitable for use in most mobile systems [10,12]. In contrast, electrostatically driven membranes offer a potential for improvement towards lower operating voltages with further miniaturization, which has not been realized so far. This is due to the inversely proportional dependence of the driving force on the electrode distance that is inherent in this type of actuation concept.

Moreover, passive valves that are commonly implemented and utilized in miniaturized diaphragm pumps reduce the possible flow rate, particularly in applications where a high flow rate is prioritized over a high backpressure resistance. This is due to the fact that a passive outlet valve can only be opened if an overpressure relative to the ambient pressure at the outlet side is present inside the pump chamber, which is high enough to overcome the mechanical restoring force of the valve in addition to the backpressure. As a result, there is a residual overpressure in the micropump relative to the ambient pressure after the fluid from the pump chamber has been ejected and the passive valve has been closed. Therefore, a certain amount of pressurized fluid remains in dead volumes inside the pump chamber, at the valves and in any transition areas. During the next priming process, this pressurized fluid expands and fills the enlarging pump chamber until the internal pump pressure has fallen far enough for the inlet valve to open. However, as the passive inlet valve can be opened only through a corresponding underpressure in the pump, the internal pump pressure must drop below the ambient pressure on the inlet side. This necessity to minimize, or even avoid, dead volumes inside the micropump additionally restricts the design freedom, which can be circumvented by applying steerable active valves on the inlet as well as on outlet side.

The scope of this work is therefore to develop a diaphragm pump with an electrostatic drive and active valves in order to overcome the obvious downsizing limit for micropumps seen in the existing publications (e.g., [12,13]) and, thus, pave the way towards the smallest possible micropump. In addition, a separate mass flow sensor is to be designed, which is fully compatible and integrable with the proposed micropump concept. The idea behind this is that, in the future, both devices can be combined easily in order to form a microdosing system that is as integrated as possible. To achieve this, our approach is to utilize a standard MEMS production process for both devices, which enables their reliable manufacturing on a microscale. Second, the devices should be conceptualized in a way that the fabrication process is as similar and compatible as possible and that they exhibit the same feature size in order to be able to be integrated easily. This could either be realized by assembling them with only a minimum of space consumption, e.g., on the opposite sides of a PCB with a through-hole, or by integrating them directly and monolithically on the same substrate, e.g., by realizing them in different technological layers. A similar approach for a microdosing system where the mass flow sensor is directly incorporated into a micropump has been already demonstrated by [43]. However, the feature sizes demonstrated there are far beyond those envisaged with the MEMS technology applied in this work, which additionally offers the potential of the full 3D integration of both devices in the future. This may then result in a controllable, self-sensing micropump with a very small footprint, which, in turn, has the potential to serve as the basis for the further development of active microfluidic systems, e.g., in environmental sensing, and for their integration into portable devices [44].

## 2. MEMS Micropump

### 2.1. The Design and Fabrication of the MEMS Micropump

**Figure 1:**
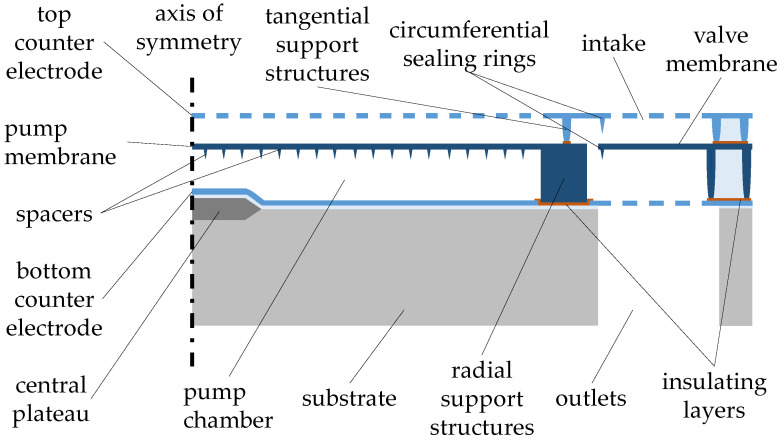
Design of the micropump: a half-cut through the device is shown, with the axis of rotational symmetry at the left side. Figure adapted from [44].

The concept of the proposed miniaturized membrane pump, as shown in Figure 1, consists of a central, bidirectional, electrostatically driven cylindrical pump chamber, surrounded by the valve area [4]. The pump membrane is suspended from radial support structures along its circumference. The spaces between those structures serve as fluidic channels, connecting the interior of the pump chamber with the valve area, thus enabling the exchange of the fluid. A small, raised plateau in the center of the pump chamber bottom reduces the actuation voltage required for the pump membrane during the ejection process without significantly reducing the available stroke volume. This design feature also ensures that the pump membrane comes into contact with the bottom counter electrode in the center first and then rolls off towards the corners, ensuring the complete expulsion of the fluid from the pump chamber [44].

The design is tailored to a low fluidic resistance inside the device in order to achieve high flow rates. To reduce the flow resistance, two primary solutions were pursued.

Firstly, actively controlled valves were used. The valve membrane is driven by electrostatic actuation in the same way as the pump membrane. However, both membranes can be moved independently from each other, using separate actuation signals. In contrast to passive valves, these active valves do not have to be opened by the positive or negative pressure inside the pump chamber, which reduces the usual problems caused by dead volumes and reduces the maximum required driving force of the pump membrane.

Secondly, the largest possible flow cross-sections were provided at the points with the highest volume flow. This was achieved, for example, by arranging the valve in a ring around the central, cylindrical pump chamber. On the one hand, this maximizes the flow cross-section in the channels between the pump chamber and the valve area, but on the other hand, it also allowed us to achieve the largest possible cross-sections for the inlet and outlet paths, respectively.

The chip plane itself separates the inlet and outlet, which facilitates integration into compact microfluidic systems by just placing the micropump directly on top of the port connecting the device to the ambient [44].

**Figure 2:**
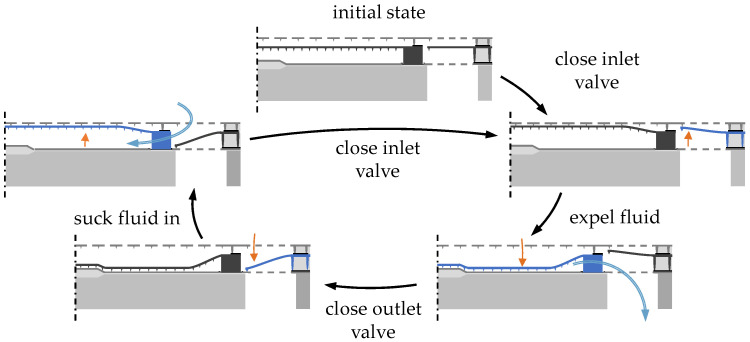
The working principle of the micropump: each stage of the pumping cycle, as well as the start-up from the resting position is depicted. Figure adapted from [44].

The working principle of the micropump is shown in Figure 2. Starting from the resting position that is depicted in the top image, both membranes are actuated in the direction of the top counter electrode, completely filling the pump chamber with fluid and closing the inlet of the micropump. In the next step, the pump membrane is pulled towards the bottom counter electrode, causing the fluid to be expelled through the open outlet. As soon as this process is complete, the valve membrane is actuated towards the bottom counter electrode, closing the outlet and connecting the pump chamber to the inlet. As the pump diaphragm is pulled up towards the top counter electrode again, the pump chamber is refilled with fluid that is sucked in from the top of the micropump chip. Then this pump cycle repeats.

Fully coupled FEM simulations accompanying the design process were used to speed up the development process of applying COMSOL Multiphysics 6.2. This is a challenging simulation task, since microfluidic devices pose extreme demands on the model and the simulation environment due to the complex interdependencies and bidirectional coupling effects between the mechanical, fluidic, and electrical energy domains, the non-linear phenomena occurring during the operation, and the large geometric aspect ratios. Therefore, the complexity of the model needs to be reduced in order to keep the computational expense low and, at the same time, to preserve the accuracy and significance of the simulation model in order to get predictive and reliable simulation results. To this end, we addressed the following aspects in deriving the simulation model:

- The complexity of the structure and the hereof resulting large finite element model;
- The bidirectional multi-energy domain coupling between the mechanical, fluidic, and electrical domains;
- The dynamically changing geometry, resulting in large mesh deformation in the fluidic and electrical energy domains;
- The mechanical contact between the pump and the valve membrane and their respective electrodes.

To address the issue of the structural complexity and the resulting, potentially very large, finite element model, the geometry was simplified to a 2D, rotationally symmetric finite element model, which matched the structure of the prototypes in a very satisfactory way. The mechanical and fluidic domains were fully coupled with each other via the corresponding multiphysics module of the software. This already constituted a strong bidirectional coupling between the structural and fluidic energy domains, meaning that a further coupling to the electrical domain, which was needed to simulate the electrostatic actuation force acting on the pump and the valve membranes, respectively, resulted either in strong convergence problems or in very long computation times. This is due to the fact that the electrical actuation influences the mechanical motion and this, in turn, by changing the electrode configuration, influences the electrostatic force. Also, calculations in the fluidic domain require a very fine mesh with extremely thin boundary layers. Solving the electrostatic equations on the same mesh came with extreme computational expenses. While setting up a separate mesh for the electrostatic domain was possible in COMSOL Multiphysics, the necessary interpolation to combine the solutions from both meshes to incorporate the domain coupling, together with positioning problems at the contact from the fluidic to the mechanical domain, made this approach unfeasible. In order to mitigate this issue, the electrical force, which varied over the membrane length due to the varying gap height between the electrodes, was analytically formulated in terms of a differential plate capacitor model, which was directly implemented into the model of the mechanical domain. The electrostatic actuation force was hence calculated as a function of the actual distance to the counter-electrodes and of the electrical voltage applied at the respective time step, and it was applied to the membrane surfaces as a position-dependent distributed load. Due to this semi-analytical approach, the full solution of the electrical field equations in the electrical domain (i.e., inside the gap regions between the pump and the valve membranes and their respective counter electrodes), as well as their couplings to the mechanical domain on a finite element basis can be avoided. This drastically decreases the simulation time and, even more importantly, the convergence problems. With this model, we are able to simulate the fluid transport induced by the moving pump membrane as a response of the micropump to an electrical control signal.

Two further challenging aspects of the modeling were the strong deformation of the computational grid (moving mesh) in the fluidic domain and the contact problem when the pump and valve membranes hit their counter-electrodes. The large deformation of the mesh inside the fluidic domain was addressed by using sophisticated meshing algorithms, which use mesh quality-based metrices to induce remeshing when necessary. The solution to the contact problem was more complex. At the bottom of the pump chamber, as well as at the upper electrodes of the micropump, there are small, point-like anti-sticking structures (see Figure 1), which create the actual contact and act as spacers. In order to model the function of these anti-sticking structures without actually having to include these fine details into the geometrical model and, this way, increasing the geometrical complexity by far, a virtual, mechanical counterforce was applied to both the pump and the valve diaphragm. This mechanical counterforce was formulated as a smoothed-out, analytical step function using the options for direct function implementation contained in COMSOL Multiphysics. In a nutshell, this force begins to become effective shortly before the non-sticking structures come into contact and then increases to a maximum value that completely stops their motion. In this way, the slightly elastic behavior of the spacers is simulated and high contact velocities, which can lead to convergence problems during the simulation, are avoided. Details on the exact formulation of this penalty function, modeling the contact force between the membranes and the small contact spacers, and its implementation can be found in [45].

**Figure 3:**
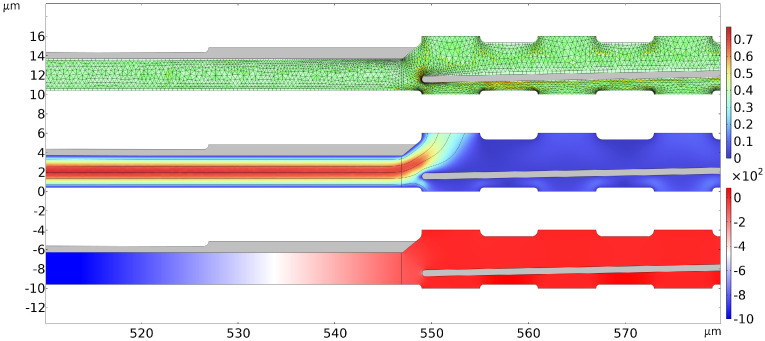
Exemplary simulation results from a time-dependent study utilizing the purpose-built FEM model. The timestep selected for the illustration shows the moment at which the valve diaphragm just touches the lower counter electrode and the suction process into the pump chamber begins. The entire computational domain is too large in the lateral direction to be displayed in a meaningful way. Thus, the image shows an enlarged view of a small section of the simulated device at the connection between the pump chamber and the valve area, the most critical part of the micropump regarding the simulation. Three different parameters are plotted in the same frame: mesh and mesh quality (**top**), fluid velocity in ms^−1^ (**center**), and gauge pressure in Pa (**bottom**).

The micropump model is meshed with a free, automatically generated triangular mesh containing approximately 5 × 10^4^ elements. The mesh refinements are generated automatically towards the edge of the fluidic domain (see Figure 3 (top)). The model was used to carry out transient FEM simulations, to enable a deeper understanding of the inner workings and interdependencies of the micropump. It is capable of supporting the design process by running parametric studies, varying the design parameters in question. An exemplary result can be seen in Figure 3. The simulations allowed us to get a deeper insight into the operation of the whole device and an understanding of the impact of the respective design parameters and enabled us to define the geometrical parameters and the design space for the prototype variants. It further helped us to identify also the weaknesses of the envisaged pump concept and to derive measures to overcome them in future designs. This has been shown by the simulation results that have been published in [41]. They reveal that the current pump concept may further be optimized by additional technological steps and geometrical optimization in order to get rid of dead volumes and to enhance the pump efficiency further. To include these findings in the here-presented devices was not possible due to the long fabrication run times of the test wafers and due to technological restrictions in the fabrication process.

**Figure 4:**
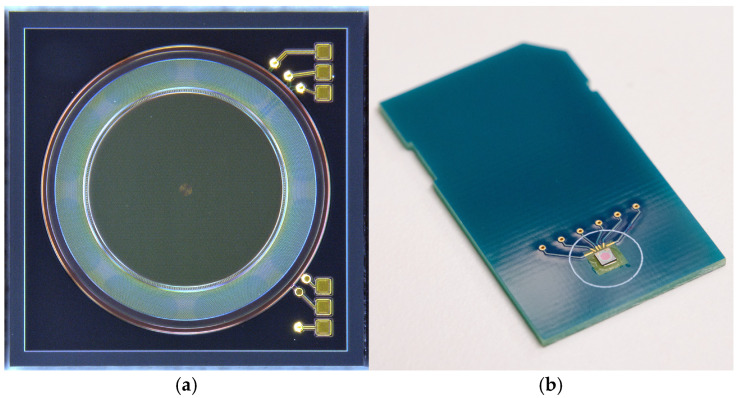
(**a**) Microscopic image of a prototype micropump chip. The chip dimensions are 1.86 mm × 1.86 mm × 0.3 mm. The pump chamber (darker, in the center) is surrounded by the valve area (lighter color). The elevated area in the center of the pump chamber floor, as well as the eight outlet ports in the substrate below the valve area, can be seen through the translucent membranes. Figure adapted from [44] (**b**) Micropump chip glued and bonded to a printed circuit board, before fitting into the protective cover. Figure adapted from [4].

Based on the preliminary investigations carried out with test structures manufactured on wafers within a similar MEMS process and the results of the above-described FEM simulations [46], novel micropump prototypes with a chip edge length of 1.86 mm (see Figure 4a) were developed and produced using a standard semiconductor manufacturing process that was similar to the one described in [47], which was basically developed for the production of MEMS microphones. In order to test a variety of process parameters and geometrical impacts, around 80 variants of the pump were designed.

### 2.2. The Characterization Setup for the MEMS Micropumps

A microscopic image of an exemplary prototype is depicted in Figure 4a. The prototypes were glued and bonded to printed circuit boards (see Figure 4b) and fitted with protective covers, which have a central port that serves as both a fluid inlet and an opening through which the optical measurements of the micropump operation can be carried out.

**Figure 5:**
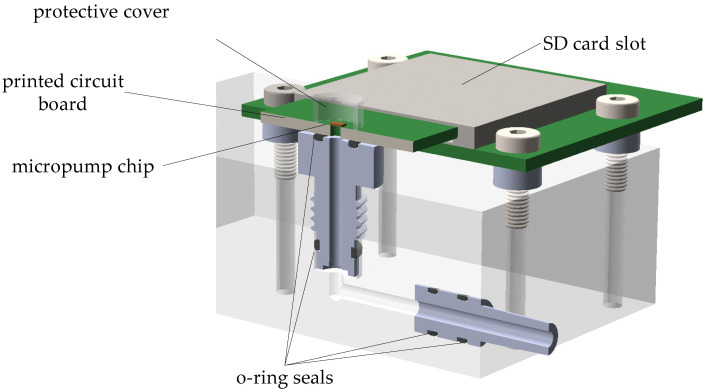
3D-cut model of the purpose-built test jig with an assembled printed circuit board on which a micropump prototype is mounted. Figure adapted from [4].

**Figure 6:**
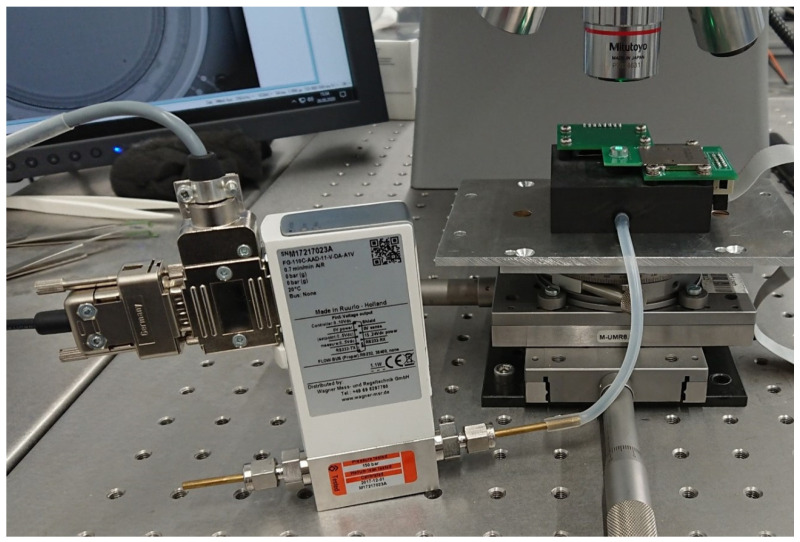
Measurement setup: Polytech MSA-500 laser Doppler vibrometer with a purpose-built test jig for prototype handling and fixation and a Bronkhorst EL-Flow Prestige mass flow meter [44].

The assembled printed circuit boards were clamped into a purpose-built test fixture (see Figure 5) via an SD card slot, which also provided the electrical contact in addition to the mechanical mounting. This made for rapid testing and the easy exchange of multiple prototypes and facilitated the handling of the otherwise very small micropump chips. The test fixture was placed on a mechanical stage underneath the microscope optics of a Polytech MSA 500 laser Doppler vibrometer for characterization. A Bronkhorst EL-Flow Prestige series mass flow sensor was used to measure the produced flow rate of each micropump (see Figure 6).

The steering signals for the micropump were provided by two Rohde & Schwarz AM300 arbitrary dual-channel function generators and amplified by an in-house, custom-built dual-channel amplifier, based on operational amplifier circuits. All the tests were performed with unfiltered ambient air.

## 3. MEMS-Based Mass Flow Sensor

### 3.1. The Design and Fabrication of the MEMS-Based Mass Flow Sensor

The mass flow sensor unites the principle of hot-wire anemometry with the miniaturization made possible by applying semiconductor manufacturing processes. Like with the micropumps, the manufacturing process used for the mass flow sensor chips is based on a standard MEMS process as well.

**Figure 7:**
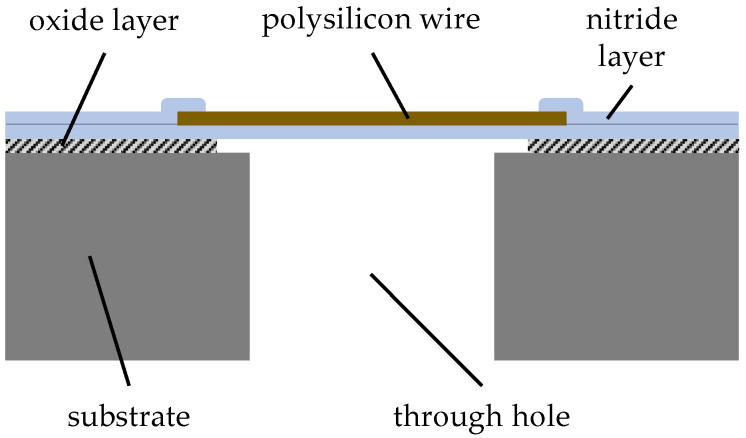
Design of the mass flow sensor. Figure adapted from [44].

The main innovation of the design is that the measured flow passes perpendicularly through the chip (see Figure 7), which was enabled by etching a through-hole through the entire substrate beneath the microstructure [1,48]. This way, the center of the heated wire, which exhibits a tapered shape to improve its robustness, was placed at the center of the mass flow to be measured and, in addition, the sense direction was aligned to the pump direction of the presented pumps, which facilitated the combination or even the integration of both.

As a supplementary design measure, a constriction of the hot wires in the center of the trough hole was introduced which increases the electrical resistance there so that most of the conversion of the electrical energy into heat takes place where the highest flow velocities are expected, i.e., in the center of the flow channel. Both factors enhance the sensitivity of the mass flow meters. In addition to the highly localized heat generation in the center of the heat wires, the heat wires are very efficiently thermally decoupled from the substrate due to the very small contact surfaces of the heat wires to the chip and the additional insulation layers, making the system less prone to external disturbances [4].

**Figure 8:**
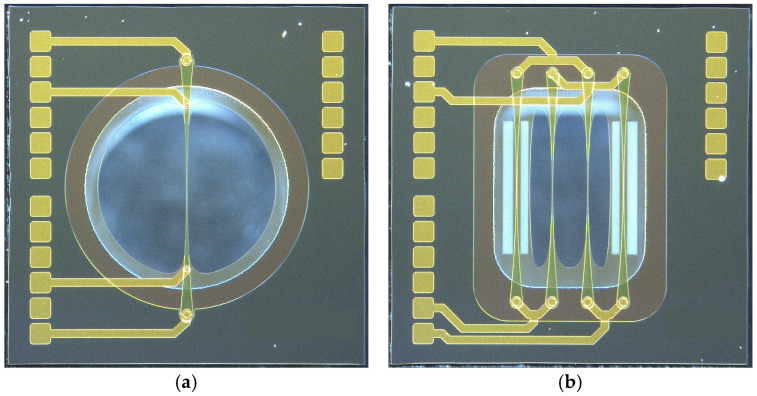
Microscopic images of silicon-based, hot-wire mass flow sensor chips. (**a**) Standard design with single wire configuration and force/sense contact pairs [44] (**b**) Improved design with a fully integrated Wheatstone bridge [44].

Different variants with one wire (see Figure 8a), as well as several wires connected to a Wheatstone bridge (see Figure 8b), were realized on chips with an edge length of 1.4 mm. Thus, the feature size of the mass flow sensors is fully compatible with the presented micropumps. The single wire variants exhibit routing for four-wire measurements and are intended as reference components for characterizing different wire shapes and material properties. The Wheatstone bridge variants comprise two wires buried in the membrane and two wires exposed to the fluid flow, arranged in an asymmetric bridge configuration to maximize the sensor signal [4].

### 3.2. Characterization Setup for the MEMS-Based Mass Flow Sensor

**Figure 9:**
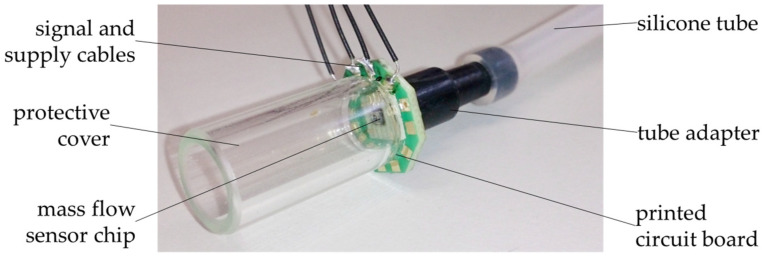
Measurement setup for the characterization of the mass flow sensor prototypes. Figure adapted from [4].

For testing, similar to the procedure for the micropumps, the chips of the mass flow sensors were assembled in custom-made, easy-to-handle prototype set-ups (see Figure 9). The chips were glued and bonded over a through-hole in a printed circuit board. On the backside, a tube adapter compatible to the Luer system was glued onto the printed circuit board to enable the attachment of medical silicone tubing.

The frontside of the printed circuit board is covered with a long, tubular, clear protective cover with an open end to allow the fluid, which is supplied through the medical tubing, to flow freely through the mass flow sensor chip and back to the ambient and, at the same time, to offer protection to the chip from external disturbances like convective airflow.

**Figure 10:**
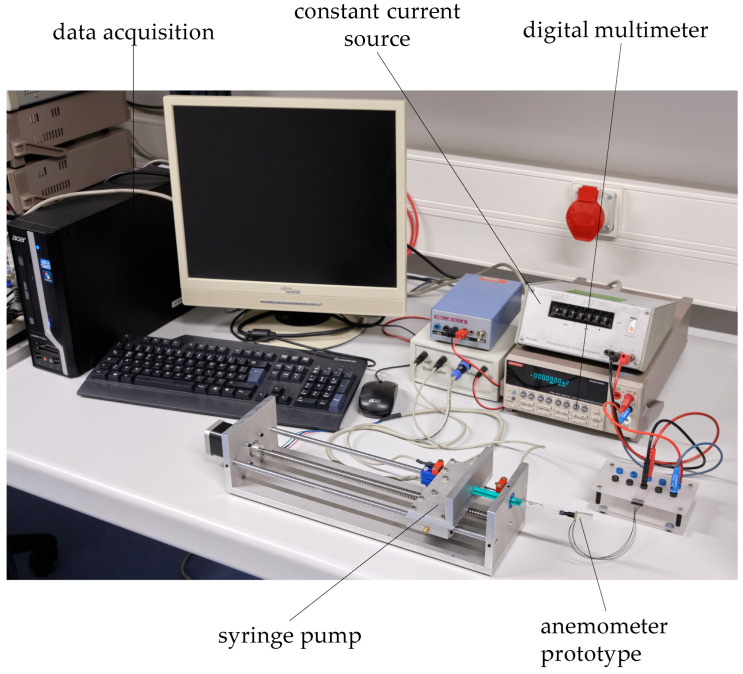
Measurement setup for the characterization of the mass flow sensor prototypes. The prototype is directly attached to the syringe. A reference sensor can be placed between the syringe and the prototype. Figure adapted from [4].

The characterization setup for the mass flow meter prototypes (Figure 10) consisted of a specially manufactured syringe pump, a reference sensor (Bronkhorst EL-FLOW^®^ Prestige series, Bronkhorst High-Tech BV, Ruurlo, The Netherlands) which was connected in series between the syringe pump and the prototypes to be tested, a constant current source to supply the prototype sensors, and a desktop multimeter to measure the sensor voltage. The syringe pump was fitted with different sizes of syringes using tubular adapters. This way, we were able to produce steady fluid flows between 80 nL/min and 23 mL/min [48]. All the tests were performed using unfiltered ambient air.

The mass flow sensor chips are characterized by a focus on showing a proof of concept of their operation and to assess their sensitivity on the pure device level. This decision for using a simple measurement method was also motivated by the fact that, for subsequent applications, a separate dedicated evaluation electronics has to be developed anyway, enabling ambient temperature and drift compensation and more complex measurement modes, such as pulsed methods or constant temperature anemometry, which are standard in the industry.

Hence, the hot wire or the bridge arrangement was simply supplied with a constant electrical current, and the voltage drop across the hot wire or the bridge arrangement was measured. A variable, distinctively adjustable volume flow was fed to the sensor through the silicone tube. Due to the temperature dependence of the electrical resistance of the hot wires, a change in the volume flow was then measured as a change in the electrical voltage that drops across the hot wire or the bridge arrangement.

## 4. Results

### 4.1. Fully Integrated Micropump

**Figure 11:**
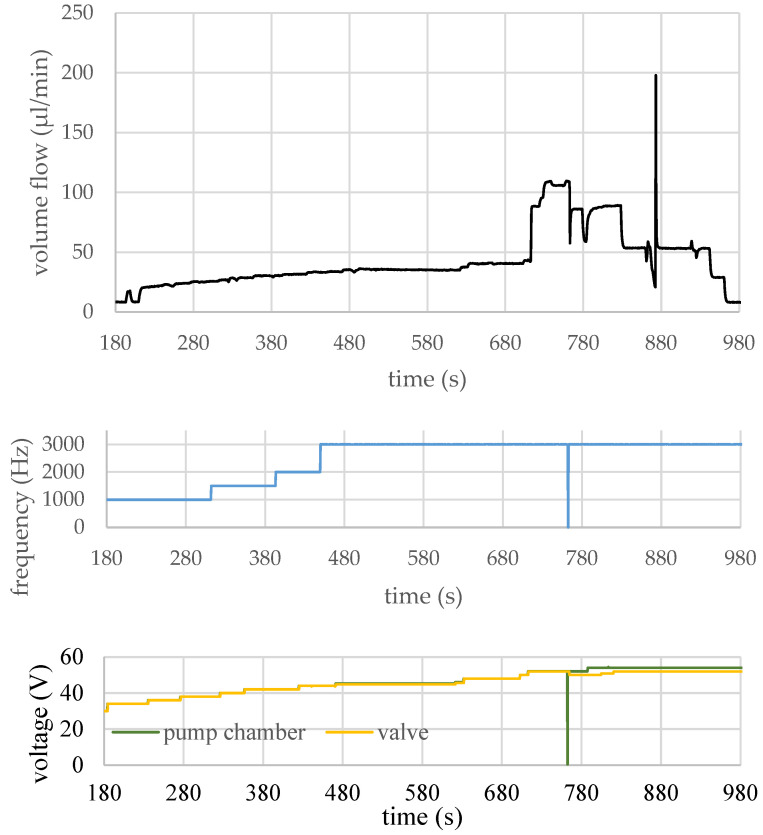
Measured volume flow: A flow rate of 110 µL/min (air) was achieved for this design (top). Additionally, the voltage and the frequency of the actuation signal (pump membrane and valve) are depicted. Figure adapted from [44].

The fully coupled FEM simulations [45] were not only used to derive the design’s space and the parameters for the prototypes but also to determine optimized parameter sets as a starting point for the control signals, which were applied for characterizing the pumps and determining their flow rates. Figure 11 shows the measurement result of a test run in which a maximum volume flow of 110 µL/min was achieved by further adjusting the control signals in terms of control voltage, operating frequency, the phase offset between the pump and the valve diaphragm, and the duty cycle of the pump and the valve control signals.

However, 762.5 s after the beginning of the experiment, the function generator creating the actuation signals briefly stalled during a change of the actuation settings, stopping the fully actuated membranes in contact with their respective counter electrodes. This caused a catastrophic electrical breakdown at the valve membrane at a supply voltage of 52 V, from which the micropump did not recover. During the remaining experiment protocol, the gradual decay of the micropump prototype can be observed. The cause for this failure was insufficient electrical insulation of the membranes, which was apparently due to the relatively high voltages applied—for the MEMS devices produced with this process.

**Figure 12:**
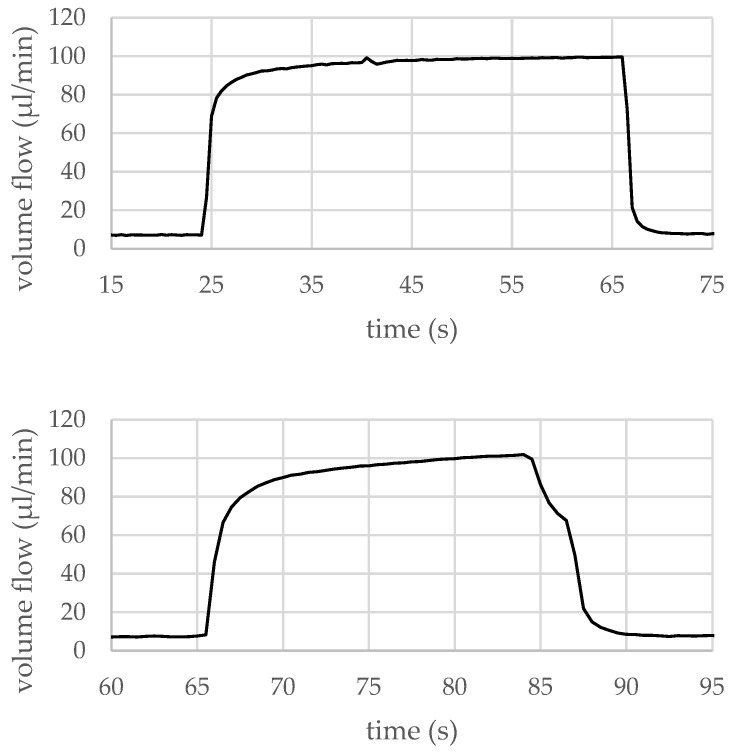
Two additional measurement protocols from experiments with other prototypes of the same type used for the previous test. Control signal parameter sets with reduced actuation voltages compared to the previous test were used. A flow rate of 100 µL/min (air) can be reliably reproduced without damaging the micropumps. Figure adapted from [4].

Continued tests with more devices revealed that, in order to get the micropumps back to a safe operating area, the supply voltages need to be reduced to a maximum of 49 V. The other control signal parameters, like the valve and pump membrane timing, were set accordingly to ensure a maximized volume flow output at an actuation frequency of 3 kHz. Multiple micropump prototypes of the same type, as used in the above-described experiment, then were tested repeatedly. The micropump prototypes were plugged into the test jig and the above-described, reduced voltage actuation signal was applied directly, without any type of soft ramp-up, neither in voltage nor in frequency. After several seconds, the actuation signal was turned off instantly and the micropumps were inspected for damages. All of the micropump prototypes tested in this way reliably reached a maximum flow rate of 100 µL/min during the experiment. Two exemplary measurement protocols that were created during those tests are shown in Figure 12, demonstrating that under these operating conditions, the prototypes can be operated safely and reliably without getting damaged.

### 4.2. MEMS-Based Mass Flow Sensor

The mass flow sensors were characterized using the measurement set-up described in Section 3.2. The sensor signals obtained for the different prototypes clearly show that the best sensor variant with the integrated measuring bridge exhibited a detection threshold of about 10 µL/min and a measuring range of up to at least 20 mL/min in the constant current mode without applying any dedicated evaluation electronics.

**Figure 13:**
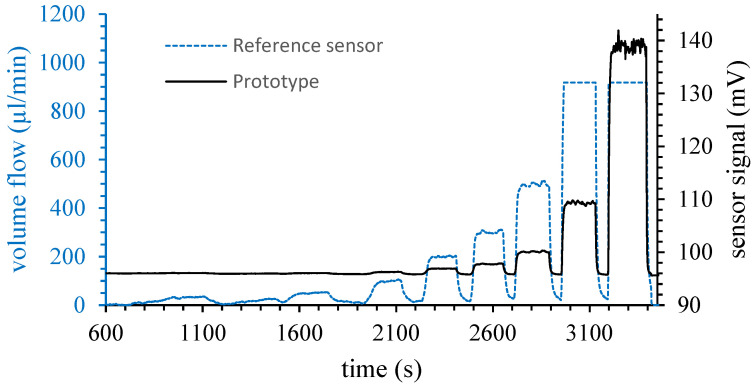
Exemplary measurement of an integrated Wheatstone bridge variant prototype compared to a reference sensor (Bronkhorst EL-FLOW^®^ Prestige series). For high flow rates the reference sensor saturates at about 917 µL/min. The last two volume flow values applied were 1600 µL/min and 2400 µL/min, respectively. Figure adapted from [44].

**Figure 14:**
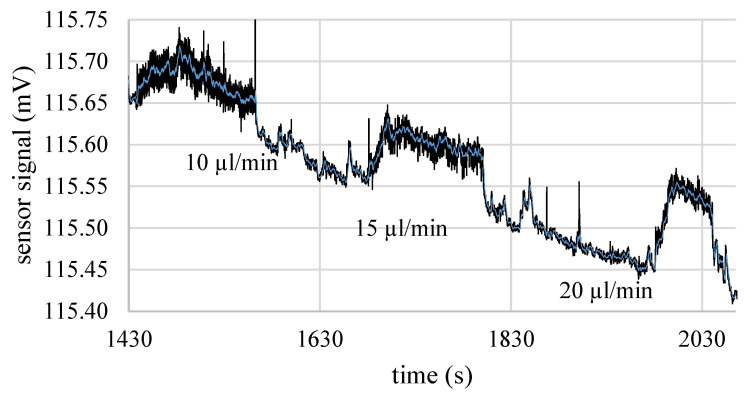
Exemplary result of an integrated Wheatstone bridge variant of the mass flow sensor, demonstrating its capability to measure very low flow rates in the range of 10 μL/min to 20 μL/min. Figure adapted from [44].

Figure 13 shows an exemplary measurement, in which it can be clearly seen that the reference sensor was already clipping the signal at about 917 µL/min, while the prototype was still able to measure the high values of the volume flow, which were 1600 µL/min and 2400 µL/min, respectively. However, when comparing the volume flow measurements from the reference sensor to the signal output of the prototype sensor, a strong non-linearity in the sensor signal can be observed. This can be seen particularly well with the two highest set values. For applications, the respective read-out electronics and a proper calibration procedure has to be applied in order to linearize the signal accordingly. However, despite the underlying signal drift, the sensitivity in the regime of small flow rates may be estimated from the diagram, since Figure 14 shows the behavior of the sensor close to its detection limit. From the course of the curve, it can be seen that the flow rate of 10 µL/min was clearly detected with a sensor signal of almost 50 µV, which is a very promising result for the sensitivity of the devices, considering the simple measurement method used. However, issues like noise, drift, and non-linearities have to be addressed in future work.

## 5. Discussion and Conclusions

This work presents a novel, miniaturized, electrostatically actuated diaphragm pump and an associated, but separate, mass flow sensor that uses the principle of hot-wire anemometry. The presented micropump and flow sensor designs are very compact compared to the state of the art and are ready for integration into mobile applications. Both devices are monolithic and manufacturable within the same standard MEMS process platform. Prototypes of both devices were tested in specially developed test benches.

At a size of only 1.86 mm × 1.86 mm × 0.3 mm, the micropumps are capable of pumping gaseous media at flow rates of up to 110 μL/min when applying an optimized parameter set for the actuation signal. The insufficient electrical insulation of the membranes leads to destruction at supply voltages above 50 V in adverse conditions. This must be addressed in further prototypes by adaptations in the design and manufacturing process to increase the breakdown voltage, by design changes to reduce the required supply voltage, or a combination of both. However, flow rates of up to 100 µL/min can already be achieved reliably, even with these prototypes.

The miniaturized mass flow sensors with chip sizes of 1.4 mm × 1.4 mm × 0.4 mm are able to detect fluid flows of around 10 µL/min up to at least 20 mL/min using a simple constant current measurement method and basic measurement equipment. The drift and non-linearities still have to be compensated for by a dedicated, application-specific integrated circuit in future work. However, well-known methods already exist for this purpose.

Both devices can, in future work, be combined in a single joint manufacturing process to form very compact, fully integrated microdosing systems. Since the pump and the sense direction both are perpendicular to the chip, this can be realized by either mounting them as a hybrid, but very compact, system on both sides of a PCB with a through-hole in between or by directly integrating the mass flow sensor functionality into the counter-electrodes of the micropumps, as they are already being produced using the same sub-process, utilizing the same material combination with similar layer thicknesses. This way, a fully integrated microdosing system can be realized, which can be easily combined with other silicon-based (electronic) components.

## 6. Patents

The European patents EP 3772589 B1 and EP 3751239 B1 resulted from the work reported in this manuscript.

## Data Availability

The original contributions presented in the study are included in the article; further inquiries can be directed to the corresponding author.
